# Anti-inflammatory effects of chlorogenic acid from *Taraxacum officinale* on LTA-stimulated bovine mammary epithelial cells via the TLR2/NF-κB pathway

**DOI:** 10.1371/journal.pone.0282343

**Published:** 2023-03-22

**Authors:** Ping Xu, Xiaobo Xu, Hanna Fotina, Tetiana Fotina

**Affiliations:** 1 School of Life Science and Basic Medicine, Xinxiang University, Xinxiang, China; 2 Faculty of Veterinary Medicine, Sumy National Agrarian University, Sumy, Ukraine; National Institutes of Health, UNITED STATES

## Abstract

Mastitis is an inflammatory disease caused by microbial infection. Chlorogenic acid (CGA), one of the major phenolic acids in *Taraxacum officinale*, has natural antioxidant and anti-inflammatory properties in various cell types; however, the effects of CGA on Lipoteichoic acid (LTA)-induced bovine mammary epithelial cells (BMECs) have not been investigated. In this study, the CGA content in *T*. *officinale* was determined by High-performance liquid chromatography (HPLC). BMECs were infected with LTA to induce the mastitis model. Different concentrations of CGA were administered after establishing the LTA infection. The results showed that the *T*. *officinale* contained CGA 1.36 mg/g. CGA significantly reduced the pro-inflammatory gene and protein expression of TNF-α, IL-6, and IL-1β. In addition, CGA downregulated the NO, TLR2, and NF-κB signaling pathways in LTA-infected bovine mammary epithelial cells. Our results indicate that CGA reduced the expression of TNF-α, IL-6, IL-1β, and TLR2 by inhibiting the phosphorylation of proteins in the NF-κB signaling pathways in a dose-dependent manner. This finding suggests that CGA may be a potential agent for the treatment of mastitis in dairy cows.

## Introduction

Mastitis is one of the most common diseases during the postpartum period in both humans and animals [1–3]. Mastitis is a global problem as it adversely affects animal health, quality of milk, and economy of milk production and every country including developed ones suffering from huge financial losses [4]. Mastitis is ranked as the most common disease in dairy cows, causing significant economic losses [5]. It was reported that the cost of mastitis treatment was about $200 per cow per year. The estimated annual cost to the US dairy industry alone is US $2 billion [6]. Because of the S. aureus infections, the loss of milk was nearly 380 tons per year in the world [7]. And *Staphylococcus aureus* (*S*. *aureus*) is the most common pathogen responsible for dairy cow mastitis [8]. The prevention and treatment of mastitis caused by *S*. *aureus* is very difficult, because *S*. *aureus* can evade the host immune system’s attack, and has the ability to hide in the host cells, making it useless for antibacterial drugs [9]. Lipoteichoic acid (LTA) is the main component of the cell wall of Gram-positive bacteria [10, 11]. Recent studies have shown that in addition to assisting *S*. *aureus* adhesion and colonization, LTA is also involved in stimulating inflammatory responses in host cells. Therefore, LTA is considered to play an important role in the pathogenesis of *S*. *aureus* [12, 13]. When *S*. *aureus* LTA invades the host cells, it induces the activation of toll-like receptor 2 (TLR2), and TLR2 can activate nuclear factor-κB (NF-κB) and mitogen-activated protein kinase (MAPK) signaling pathways, resulting in the significant secretion of inflammatory cytokines [14, 15].

Historically, plants have been a rich source of antibacterial, antiviral, and immunomodulatory metabolites. *Taraxacum officinale* (*T*. *officinale*) is a famous traditional Chinese herb that is commonly used for the treatment of inflammatory and infectious diseases, such as respiratory tract infection, hepatitis, bronchitis, mastopathy, and pneumonia [16–18]. Some plant extracts have been used for treating clinical mastitis on organic dairy farms with good therapeutic effect [19]. Chlorogenic acid (CGA), isolated from *T*. *officinale*, has been shown anti-inflammatory [20], antibacterial [21], anti-viral [22], and anti-oxidative [23] effects in several studies. A previous study demonstrated that CGA alleviated the inflammatory response induced by *E*. *coli* in sheep endometrial epithelium cells via inhibiting activation of the TLR4/NF-κB signaling pathway [24]. Furthermore, CGA was found to be a potential therapeutic compound for LPS-induced bovine mastitis, via reduction of NF-κB [25]. However, no studies have investigated the effects of CGA for the treatment of bovine mastitis caused by LTA. Thus, the aim of this work was to examine the anti-inflammatory effects of chlorogenic acid in *S*. *aureus* LTA-stimulated BMECs and to investigate potential mechanisms for these effects.

## Materials and methods

### Ethics statement

All experimental plant procedures were comply with the IUCN Policy Statement on Research Involving Species at Risk of Extinction and the Convention on the Trade in Endangered Species of Wild Fauna and Flora. All experimental cell procedures were approved by the Animal Care and Use Committee of the Sumy National Agricultural University, Sumy, Ukraine, and the Xinxiang University, Xinxiang, China, and performed in accordance with the animal welfare and ethics guidelines.

### Data availability

The datasets analysed during the current study are available in the NCBI repository. The direct link and accession numbers were in Table 1.

**Table 1:** Quantitative real-time PCR primer information.

Gene	Accession Number	Direct Link	Sequence	Product size (bp)
TNF-α	NM_173966.3	https://www.ncbi.nlm.nih.gov/nuccore/NM_173966.3/	F:5’GGTGGTGGGACTCGTATGCCAATGC3’ R:5’GTGAGGAACAAGGGGGTGG3’	151
IL-6	NM_173923.2	https://www.ncbi.nlm.nih.gov/nuccore/NM_173923.2/	F:5’ACAGCTATGAACTCCCGCTT3’ R:5’ TCTCACATATCTCCTTTCTCATTGC3’	226
IL-1β	XM_005889988.2	https://www.ncbi.nlm.nih.gov/nuccore/XM_005889988.2 /	F:5’TCGAAACGTCCTCCGACGAG3’ R:5’ TGAGAGGAGGTGGAGAGCCT3’	131
GADPH	NM_001034034.2	https://www.ncbi.nlm.nih.gov/nuccore/NM_001034034.2/	F:5’AGATGGTGAAGGTCGGAGTG3’ R:5’CGTTCTCTGCCTTGACTGTG3’	189

### Plant material

Whole *Taraxacum officinale* plants were collected from the campus of Sumy National Agrarian University, Sumy, Ukraine, in May 2019 and were identified by Professor Li MENG, Henan Institute of Science and Technology, Xinxiang, China. The verified specimen was stored at the Institute of Chinese Materia Medica, Henan University (Kaifeng, Henan, China). The whole plants were cleaned, dried (moisture content 82%) in the shade for several days, and pulverized in a laboratory crusher.

### Reagents

Reference substance of chlorogenic acid (the standard curve for calculation purity ≥ 98%) was provided by Sigma-Aldrich (USA, Louis, MO, No 110885–200102). Acetonitrile and methanol (HPLC grade, 99.9%) were purchased from Fisher (USA, Louis, MO). HPLC-grade water was made by double-distilling pre-deionized water. All other reagents were analytically pure.

### High-performance liquid chromatography (HPLC) analysis [26]

#### Preparation of *T*. *officinale* extracts

One gram of sample powder was accurately weighed and 50 mL of 60% ethanol extraction solvent was added. The sample then underwent ultrasonic (power 250 W, frequency 35 KHz) extraction for 40 min, followed by suction filtration. The extraction operation was repeated three times and the extracts were combined, and concentrated under reduced pressure at 50°C using a rotary evaporator (N-1300D, EYELA, Japan). Finally, the extracts were diluted up to 25 mL with extraction solvent. The sample solutions were stored at 4°C. Sample solutions were filtered using a 0.45 μm filter before injection.

#### Chromatographic conditions and instrumentation

Analysis was performed on a Shimadzu Acquity HPLC system (LC-20A, Shimadzu, Japan). An Inertsil ODS-3 C18 column (5 μm, 250*4.6 mm) was applied for all analyses. The mobile phase was composed of A (MeOH) and B (1‰ Phosphoric acid solution, adjusted to pH 8.0 with ammonia-water) with a gradient elution: 0–5 min, 5% A; 5–15 min, 5–15% A; 15–20 min, 15%–5% A; 20–25 min 5% A. The flow rate of the mobile phase was 1.0 mL/min. The column temperature was maintained at 30°C. The detection wavelength was set at 350 nm. The target peak was identified by comparing their retention time to that of the respective standard. A standard graph of chlorogenic acid was prepared by plotting concentration versus peak area.

#### Standard preparation

A 10 mg chlorogenic acid standard was weighed, and dissolved in a 10 mL one-mark volumetric flask with 10% methanol to form a 1 mg/mL stock solution. Methanol 10% was added to 5 mL of 1 mg/mL chlorogenic acid solution, to a constant volume of 10 mL. The solution was then sequentially diluted to obtain 6 concentrations of the reference mixture: 1, 0.5, 0.25, 0.125, 0.0625, and 0.0313 mg/mL chlorogenic acid standard solution. The standard solutions were filtered through a 0.45 μm membrane prior to injection. All solutions were stored at 4°C before analysis.

#### Cell culture and treatment

Bovine mammary epithelial cells were isolated by our laboratory from mid-lactation dairy cow milk. Briefly, the BMECs were cultured in Dulbecco’s modified Eagle’s medium/Nutrient Mixture F-12 (DMEM/F12) (Gibco, USA, New York, cat. 12400–024) supplemented with 10% fetal bovine serum (FBS) (Biological Industries, Israel, Kibbutz Beit-Haemek, cat. 04-011-1A/B) and 10 ng/mL epidermal growth factor (EGF) (Sigma, USA, St. Louis, MO, cat. E4127). Cells were maintained in an incubator at 37°C and 5% CO_2_. Cells were routinely passaged at a rate of 70–80% for all experiments. Cells were pretreated with different concentrations of CGA (25, 50, 100 ng/μL) for 5 h, followed by incubation with 20 ng/μL lipoteichoic acid (LTA) (Inviogen, Carlsbad, CA, USA, cat. tlrl-slta) for 24 h. Phosphate buffer saline (PBS) was used as a positive control. Total protein, supernatant, and mRNA were extracted from cells at specified time intervals.

#### CCK-8 assay of cell viability

The cell viability of CGA on BMECs were determined using the Cell Counting Kit-8 (cck-8, Solarbio Science & Technology Co., Ltd., Beijing, P. R. China, cat. CK04) in accordance with the manufacturer’s instructions. The cells were seeded at a concentration of 1x10^4^ cells per well in 96-well plates with eight replicates per condition. The cells were stimulated with CGA (25, 50, 100 μg/mL) and then treated with LTA (20 ng/μL) for 24 h. CCK-8 solution, at a dilution of 1:10, was added to each well at the indicated timepoint and the plate was incubated at 37°C for 3 h. Finally, the absorbance was measured at a wavelength of 450 nm using a microplate reader.

#### Quantitative real-time PCR analysis

Total RNA was extracted from the cells using RNAiso Plus (TaKaRa, Dalian, P. R. China, cat. 9109) in accordance with the manufacturer’s instructions. The assessment of the quantity and quality of RNA was verified using a NanoDrop 1000 (Thermo Scientific, Co., Ltd., P. R. China). The 260: 280 nm optical density value was between 1.8 and 2.0. The first-strand cDNA was synthesized using a PrimeScript^TM^ RT reagent Kit with gDNA Eraser (TaKaRa, Dalian, P. R. China, cat. RR047A). Quantitative real-time PCR was performed using TB Green Premix Ex Taq^TM^ II (TaKaRa, Dalian, P. R. China, cat. RR820B) on a 7500 Real-Time PCR system (Applied Biosystems Inc., Foster City, CA, USA). GAPDH was used as the reference gene. The relative gene expression was calculated using the 2^-△△Ct^ method. The primers were designed using Primer Premier 5.0 and synthesized by Sangon Biotech (Shanghai, P. R. China, Co., Ltd.). The primers used are listed in Table 1.

### Enzyme-linked Immunosorbent Assay (ELISA) [27]

The BMECs were pre-treated with CGA 5 h before LTA stimulation, and the supernatants were then collected 24 h later. The concentrations of TNF-α, IL-6, and IL-1β in the supernatants were measured using ELISA kits (Jiangsu Mei Biao Biological Technology Co., Ltd., Jiangsu, P. R. China, cat. MB-4838A\MB-4905A\MB-4837A).

### NO assay [28]

The cells were pre-treated with CGA 5 h before LTA stimulation for 24 h after which the supernatants were collected. The concentration of NO in the supernatants was measured using a Griess reagent Nitric Oxide Assay Kit (Beyotime Biotechnology, Shanghai, P. R. China, cat. S0023) via detection of the nitrite level as the nitrite level represents NO production.

### Western blot analysis [28]

The whole cell proteins were isolated using RIPA buffer containing a protease inhibitor mixture. The concentration of protein in the extract from BMECs was determined using a BCA Protein Assay Kit (Solarbio Science & Technology Co., Ltd., Beijing, P. R. China, cat. PC0020). Then, 40 μg proteins were separated on 12% SDS-PAGE, transferred onto PVDF membranes, and blocked with 5% non-fat dry milk in TBST for 2 h. The membranes were then incubated with primary antibodies overnight at 4°C and then incubated with HRP-conjugated secondary antibodies. β-Actin was used as the control. The protein levels of specific target genes expressed by electrophoresis were detected with a 3,3’-diaminobenzidine (DAB) substrate chromogenic assay (n = 3).

### Statistical analysis

Analysis was performed using GraphPad Prism 7.02 software (GraphPad Software, Inc., USA). Differences between mean values of normally distributed data were analyzed using one-way analysis of variance (ANOVA). A p<0.05 or p<0.01 was considered statistically significant. All data were obtained from three independent experiments.

## Results

### High-performance liquid chromatography (HPLC) analysis

**Fig 1:**
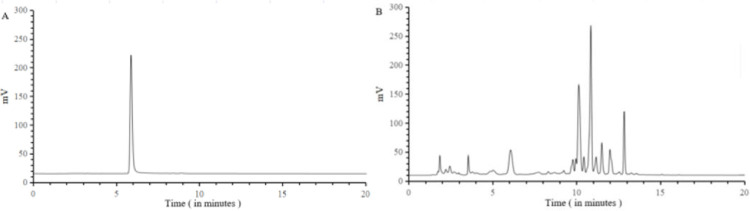
Results of high-performance liquid chromatogram of the chlorogenic acid. (A) HPLC of the chlorogenic acid standard, and (B) HPLC of the chlorogenic acid in dandelion sample.

The high-performance liquid chromatogram for chlorogenic acid standard is shown in Fig 1. The peak shape was good using this method. The detection object and other components were well separated. The standard curve for chlorogenic acid was y = 1.4624 x −2.5424, the linear range was 0.00692 ~ 0.44 μg/mL and the linear relationship was good (R^2^ = 0.999). The dandelion sample was detected using this method, and the high-performance liquid chromatogram of the sample is shown in Fig 1. By generating the standard curve for calculation, the CGA content in sample was measured to be 1.36 mg/g.

### Effects of CGA on cell viability

**Fig 2:**
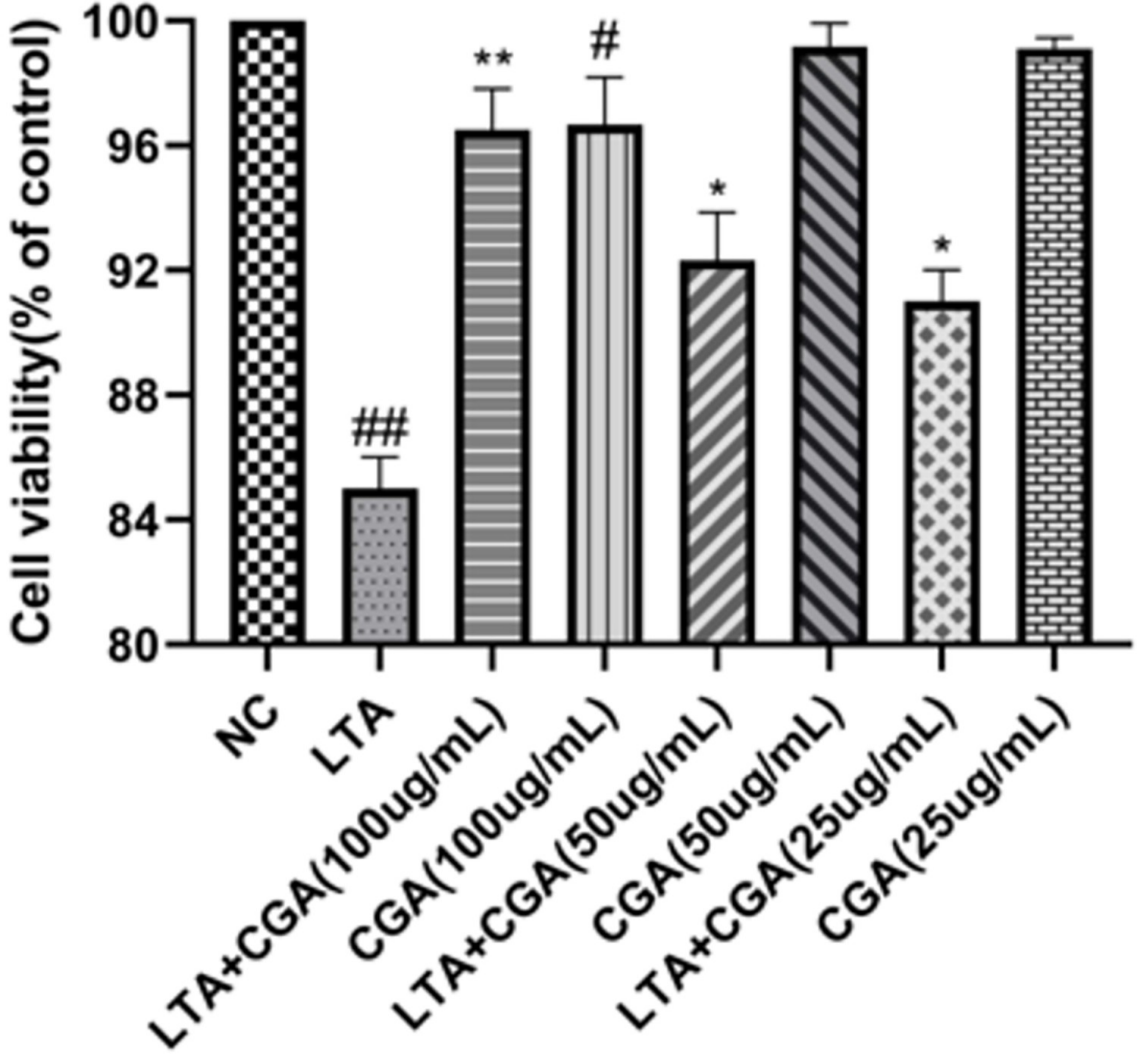
The effect of chlorogenic acid (CGA) on the cell viability of bovine mammary epithelial cells (BMECs). The values presented are the mean ± SE of three independent experiments. ## p < 0.01 vs. control group; * p < 0.05, ** p < 0.01 vs. LTA group. NC: normal control; LTA: lipoteichoic acid; LTA+CGA: lipoteichoic acid and chlorogenic acid.

CGA cytotoxicity on cell viability was evaluated by Cell counting kit-8 (CCK-8) assay after incubating BMECs for 24 h. Establishment of an inflammation model by treating BMECs with 20 ng/mL LTA for 24 hours [29]. As shown in Fig 2, the results showed that CGA (25, 50, and 100 μg/mL) had no cytotoxic effects on BMECs. LTA has an inhibitory effect on cell viability compared to NC, and CGA could abrogate LTA-induced decrease of cell viability at the dose of 25, 50, and 100 μg/mL.

### Effects of CGA on inflammatory cytokines

**Fig 3:**
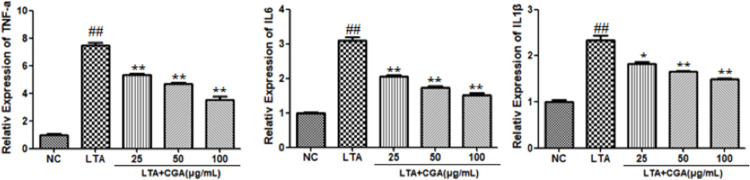
Effect of chlorogenic acid (CGA) on lipoteichoic acid (LTA)-induced gene expression of inflammatory cytokines. The values presented are the mean ± SE of three independent experiments. ## p < 0.01 vs. control group; * p < 0.05, ** p < 0.01 vs. LTA group. NC: normal control; LTA: lipoteichoic acid; LTA+CGA: lipoteichoic acid and chlorogenic acid.

**Fig 4:**
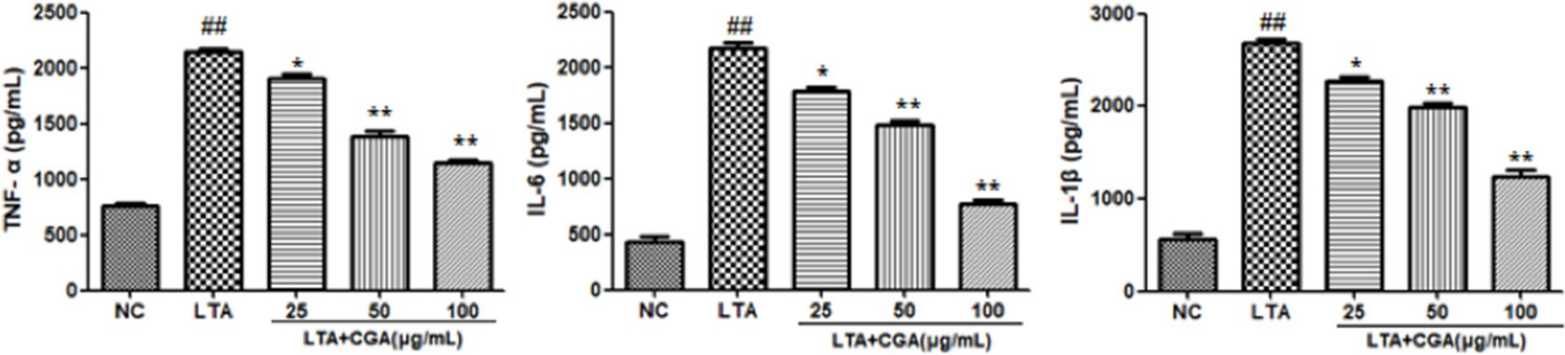
Effect of chlorogenic acid (CGA) on lipoteichoic acid (LTA)-induced protein expression of inflammatory cytokines. The values presented are the mean ± SE of three independent experiments. ## p < 0.01 vs. control group; * p < 0.05, ** p < 0.01 vs. LTA group. NC: normal control; LTA: lipoteichoic acid; LTA+CGA: lipoteichoic acid and chlorogenic acid.

To investigate the anti-inflammatory effects of CGA, the levels of pro-inflammatory cytokines were detected by qRT-PCR, and the expression of inflammatory factors in cell supernatants was measured by ELISA. As shown in Figs 3 and 4, LTA significantly upregulated TNF-α, IL-6, and IL-1β gene and protein expression compared to control. CGA increased TNF-α, IL-6, and IL-1β gene and protein expression. However, CGA suppressed TNF-α, IL-1β, and IL-6 gene and protein expression in LTA-stimulated BMECs in a concentration dependent manner.

### Effects of CGA on Nitric oxide (NO) production

**Fig 5:**
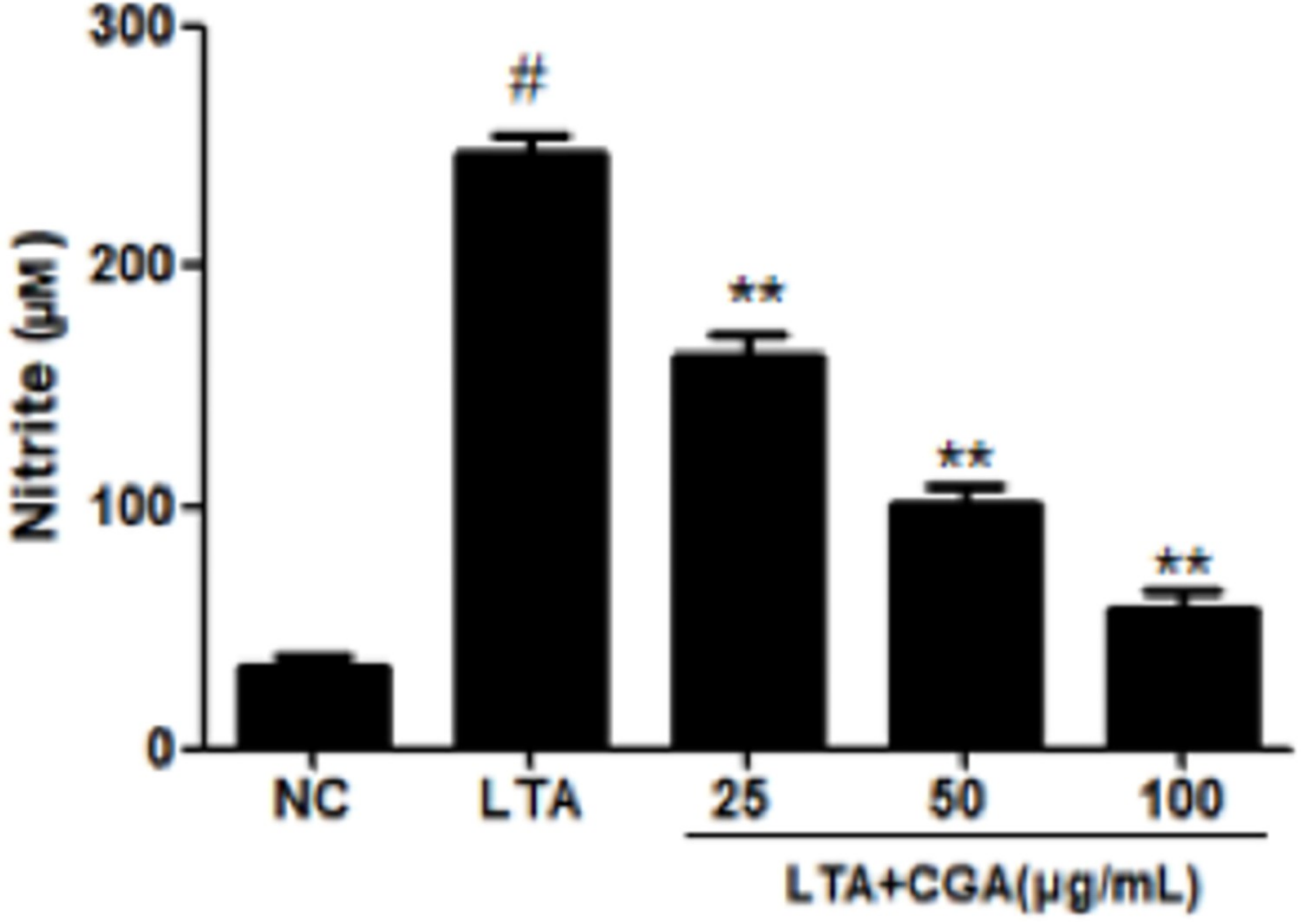
Effect of chlorogenic acid (CGA) on Nitric oxide (NO) production. The values presented are the mean ± SE of three independent experiments. # p < 0.05 vs. control group; ** p < 0.01 vs. LTA group. NC: normal control; LTA: lipoteichoic acid; LTA+CGA: lipoteichoic acid and chlorogenic acid.

The production of NO is caused by the continuous expression of iNOS. The effects of CGA on NO production induced by LTA were measured (Fig 5). The results showed that NO production in BMECs was significantly increased after LTA stimulation, and CGA inhibited the increase of NO production in a concentration-dependent manner.

### Effects of CGA on LTA-induced TLR2 expression

**Fig 6:**
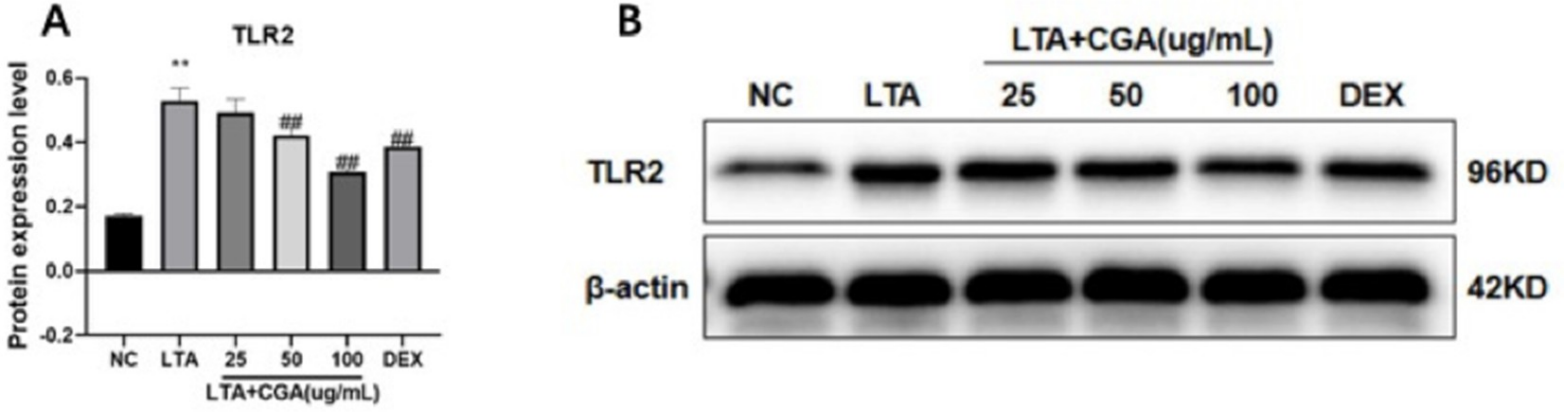
Effect of chlorogenic acid (CGA) on toll-like receptor 2 (TLR2) expression. The expression of TLR2 in BMECs was analyzed via Western blot. β-actin was used as a reference control. Dexamethasone (DEX) was used as a positive control. (A) The quantification histogram of TLR2 protein expression normalized by β-actin. The values presented are the mean ± SE of three independent experiments. ** p < 0.01 vs. control group; ## p < 0.01 vs. LTA group. (B) The expression levels TLR2. Original blots are presented in S1, S2 Figs in S1 Raw images. NC: normal control; LTA: lipoteichoic acid; LTA+CGA: lipoteichoic acid and chlorogenic acid; DEX: Dexamethasone.

The anti-inflammatory mechanism of CGA was further studied by detecting the expression of TLR2 by Western blot. The results showed that the expression of TLR2 was significantly up-regulated with LTA treatment. Meanwhile, pretreatment with CGA significantly inhibited LTA-induced TLR2 expression by a dose-dependent manner (Fig 6).

### Effects of CGA on NF-κB signaling pathway activation

**Fig 7:**
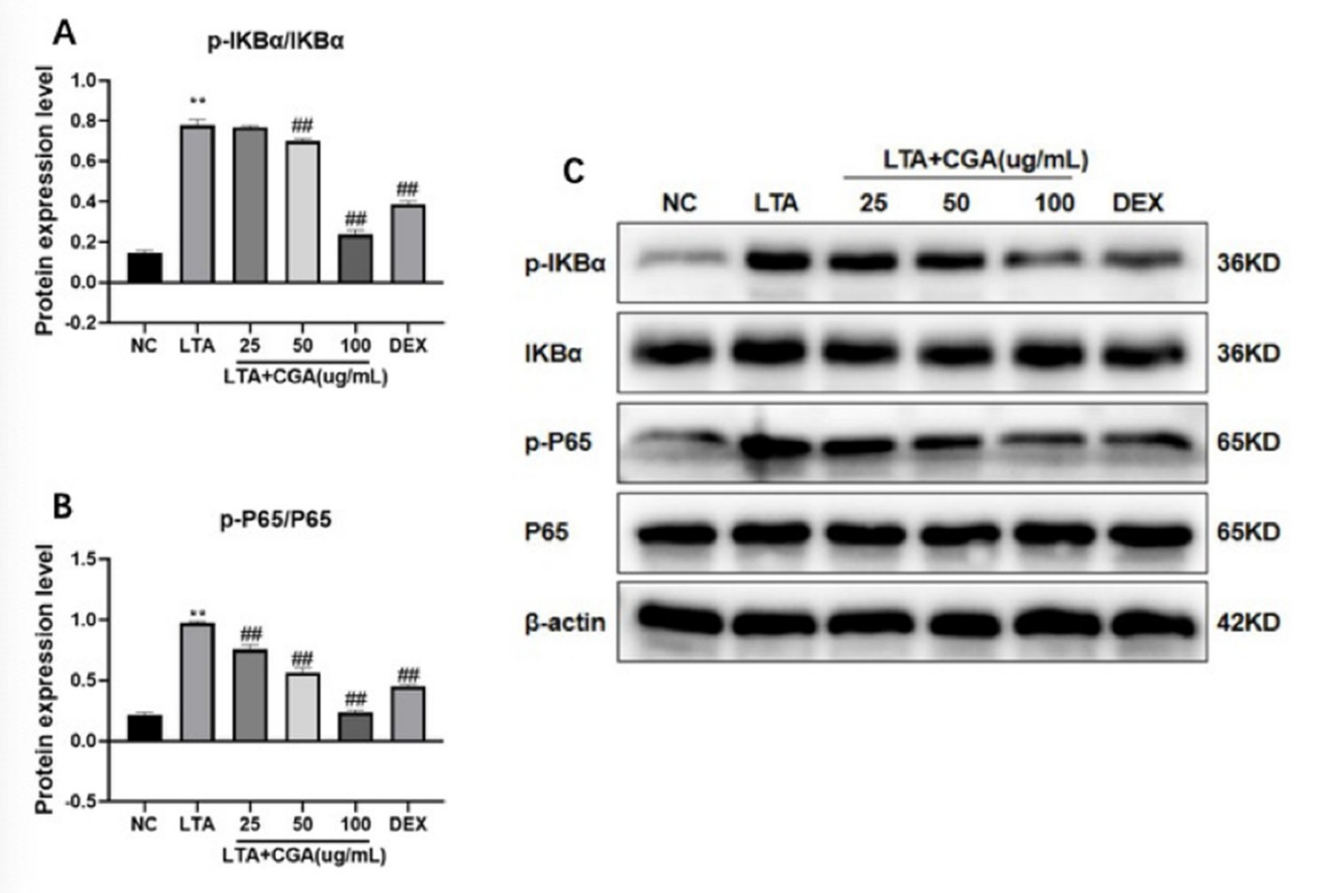
Effect of chlorogenic acid (CGA) on nuclear factor-κB (NF-κB) activation. The NF-κB pathway was analyzed in BMECs via Western blot. β-actin was used as a reference control. Dexamethasone (DEX) was used as a positive control. (A) The quantification histogram of p-IκBα protein expression normalized by β-actin; and (B) the quantification histogram of p-p65 protein expression normalized by β-actin. The values presented are the mean ± SE of three independent experiments. **p < 0.01 vs. control group; ##p < 0.01 vs. LTA group. (C) The expression levels of p-IκBα, IκBα, p-p65, and p65. Original blots are presented in S3-S7 Figs in S1 Raw images NC: normal control; LTA: lipoteichoic acid; LTA+CGA: lipoteichoic acid and chlorogenic acid; DEX: Dexamethasone.

Whether NF-κB is involved in the mechanism of CGA regulating inflammatory response was investigated by assessing the phosphorylation status of IkBα and p65. The results showed that the NF-κB signaling pathway was significantly up-regulated after LTA treatment. However, with CGA pretreatment, the phosphorylation of IkBα and p65 in LTA-stimulated BMECs (Fig 7) was significantly inhibited. Meanwhile, the results also indicated that CGA inhibited LTA-induced NF-κB activation in a dose-dependent manner.

## Discussion

Mastitis, an inflammation of the mammary gland, is a serious disease triggered in cattle by infection with bacteria, which results in decreased milk production and quality [30]. The inflammatory response to bovine mastitis caused by *S*. *aureus* infection is often slow, and the infection causes both subclinical mastitis and clinical mastitis [31]. A previous study demonstrated that lipid-rich extract from avocado seed reduces *S*. *aureus* internalization into BMECs and regulates TAP, BNBD5, and DEFB1 gene expression [32]. However, the molecular mechanism of chlorogenic acid (CGA) in *S*. *aureus* LTA-stimulated BMECs remains unclear. In the present study, BMECs were stimulated by *S*. *aureus* LTA with or without CGA pretreatment, and the effect of CGA on the inflammatory response was investigated.

Dandelion (*Taraxacum officinale*) has a long history of use in the treatment of human diseases including inflammation [33]. Dandelion extracts may reduce nitric oxide (NO), prostaglandin (PG) E2, TNF-α, IL-1β, IL-6 and cyclooxygenase (COX)-2 in the body, thereby affecting macrophages, exert an anti-inflammatory effect [34–36]. Chlorogenic acid, a phenolic compound formed by the esterification of trans-cinnamic acid and quinine, is the main medicinal component of dandelion and plays a key role in antibacterial and anti-inflammatory effects [37]. Results of our study indicate that CGA does not have cytotoxicity to BMECs and CGA can enhance viability of BMECs challenged with LTA. These results agree with those of another study demonstrating that CGA has antimicrobial activity against *S*. *aureus* and *E*. *coli* [38].

Cytokines, such as TNF-α, IL-1β, and IL-6, play a crucial role in inflammatory process [39]. They are the major markers of inflammation and are involved in host defense against inflammatory diseases. Previous studies have shown that TNF-α, IL-1β, and IL-6 are closely related to mastitis pathogenesis, and expression of these three inflammatory cytokines are significantly increased in mastitis caused by *S*. *aureus* infection [39–42]. Hu et al. [24] demonstrated that CGA suppressed the expression of TNF-α, IL-1β, and IL-6, and significantly inhibited *Escherichia coli*-induced inflammation in sheep endometrial epithelium cells. Ali et al. [43] found that CGA has a hepatoprotective effect via attenuating pro-inflammatory and apoptotic mediators and improving antioxidant competence in hepatic tissues. Similar to previous studies, pretreatment with CGA significantly decreased the expression of three pro-inflammatory cytokines (TNF-α, IL-1β, and IL-6) in LTA-stimulated BMECs in our study.

TLR2 plays a key role in the innate immune response to *S*. *aureus* infection [44]. LTA, a bacterial endotoxin embedded in the cytoderm of *S*. *aureus*, activates inflammatory responses [45] and TLR2 is the major receptor activated in response to LTA. Activation of TLR2 leads to activation of NF-κB, which regulates the expression of inflammatory cytokines [46]. Result of this study showed that CGA significantly inhibited *S*. *aureus* LTA-induced TLR2 expression, suggesting that CGA inhibits *S*. *aureus* LTA-induced inflammation by suppressing the TLR2 signaling pathway. Furthermore, *S*. *aureus* infection activates TLR2, in turn activating the downstream NF-κB signaling pathways, thus inducing significant secretion of inflammatory cytokines, eventually causing severe inflammation in mammary glands and cells [12, 47]. The transcription factor NF-κB is a key regulator of inflammatory and immune responses [48]. Cells are treated with various inducers, resulting in the degradation of IκBα; then the location of phosphorylated NF-κB p65 is transferred from the cytoplasm to the nucleus, and finally the transcription of inflammatory cytokines is promoted [49]. A previous study showed that internalization of *S*. *aureus* was associated with the active state of NF-κB, and that inhibition of NF-κB activation could attenuate the BMEC internalization of *S*. *aureus* [50]. In the present study, LTA caused phosphorylation of NF-κB p65 and IκBα in BMECs. Further studies found that LTA-induced NF-κB activation could be inhibited by CGA in a dose-dependent manner. These results suggest that CGA attenuates bacterial toxin-induced inflammatory responses by inhibiting NF-κB activation.

In our study, we demonstrated that *T*. *officinale* contained 1.36 mg/g CGA. CGA significantly reduced the inflammatory cell gene and protein expression of TNF-α, IL-6, IL-1β, and TLR2, dose-dependently, by inhibiting the phosphorylation of proteins in the NF-κB signaling pathways in LTA-infected bovine mammary epithelial cells. This finding suggests that CGA may be a potential agent for the treatment of mastitis in dairy cows. Then, we will further study on the anti-inflammatory effect of CGA in vivo. As an extract based on traditional Chinese medicine, CGA’s anti-inflammatory effects on LTA-induced mastitis in mice will provide a theoretical basis for the clinical treatment of bovine mastitis in the years to come.

## Supporting information

S1 Raw images (PDF)
