# Supplementary material for: Anti-inflammatory effects of chlorogenic acid from Taraxacum officinale on LTA-stimulated bovine mammary epithelial cells via the TLR2/NF-κB pathway
Source: PLoS One. 2023 Mar 22;18(3):e0282343. doi: 10.1371/journal.pone.0282343 (PMC10032541; doi:10.1371/journal.pone.0282343)
Supplement: S1 Raw images — (PDF) [file pone.0282343.s001.pdf]

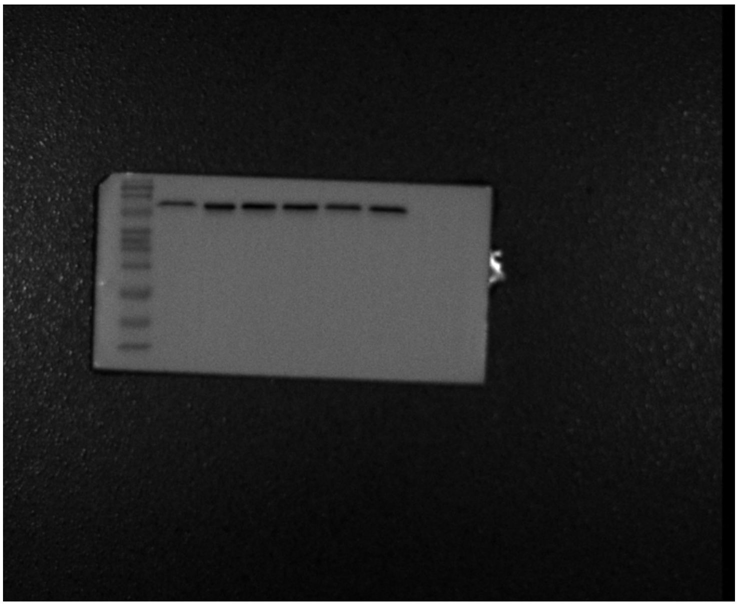

Figure 6. Effect of chlorogenic acid (CGA) on toll-like receptor 2 (TLR2) expression. The expression of TLR2 in BMECs was analyzed via Western blot.

Note: Marker, NC, LTA, LTA+CGA (25 ug/mL), LTA+CGA (50 ug/mL), LTA+CGA (100 ug/mL), Dexamethasone (positive control).

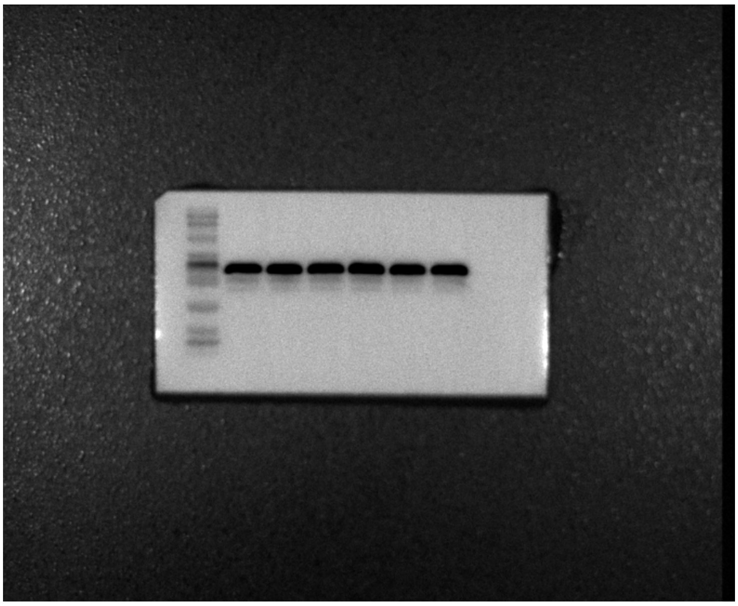

Figure 6. Effect of chlorogenic acid (CGA) on toll-like receptor 2 (TLR2) expression. β-actin was used as a reference control.

Note: Marker, NC, LTA, LTA+CGA (25 ug/mL), LTA+CGA (50 ug/mL), LTA+CGA (100 ug/mL), Dexamethasone (positive control).

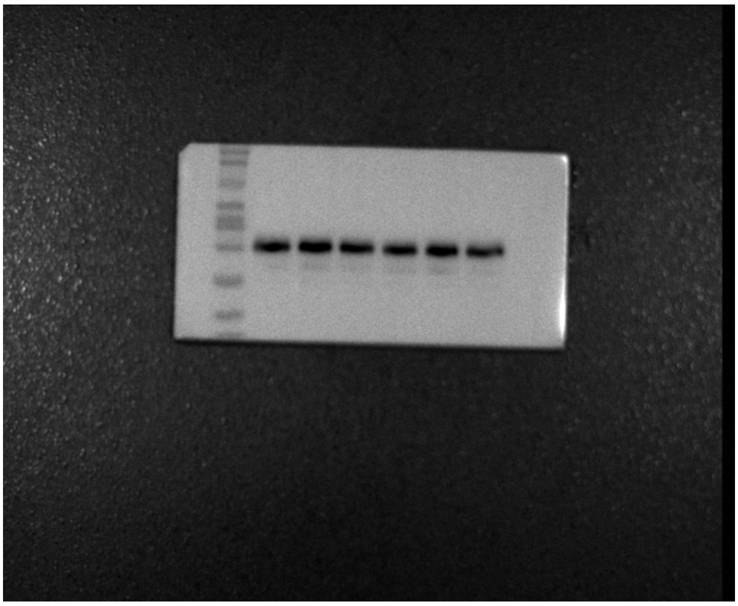

Figure 7. Effect of chlorogenic acid (CGA) on nuclear factor-κB (NF-κB) activation. The NF-κB pathway p-IKBα protein expression was analyzed in BMECs via Western blot.

Note: Marker, NC, LTA, LTA+CGA (25 ug/mL), LTA+CGA (50 ug/mL), LTA+CGA (100 ug/mL), Dexamethasone (positive control).

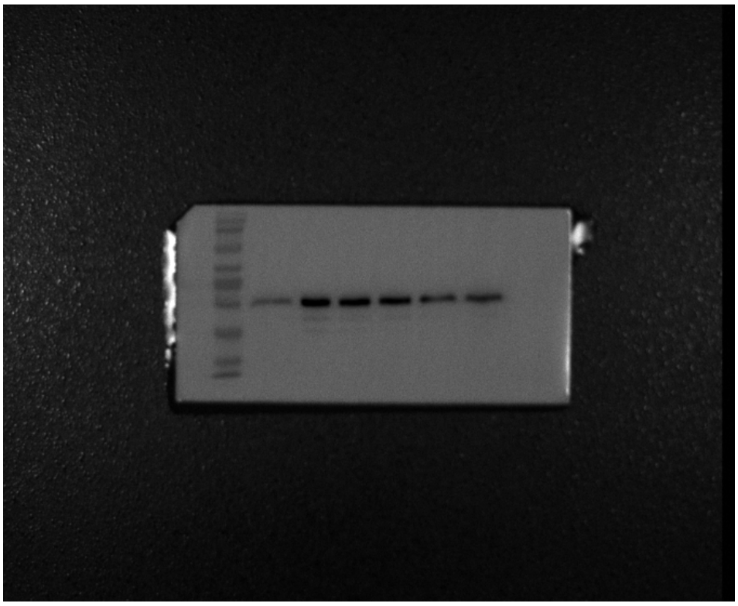

Figure 7. Effect of chlorogenic acid (CGA) on nuclear factor-κB (NF-κB) activation. The NF-κB pathway IKBα protein expression was analyzed in BMECs via Western blot.

Note: Marker, NC, LTA, LTA+CGA (25 ug/mL), LTA+CGA (50 ug/mL), LTA+CGA (100 ug/mL), Dexamethasone (positive control).

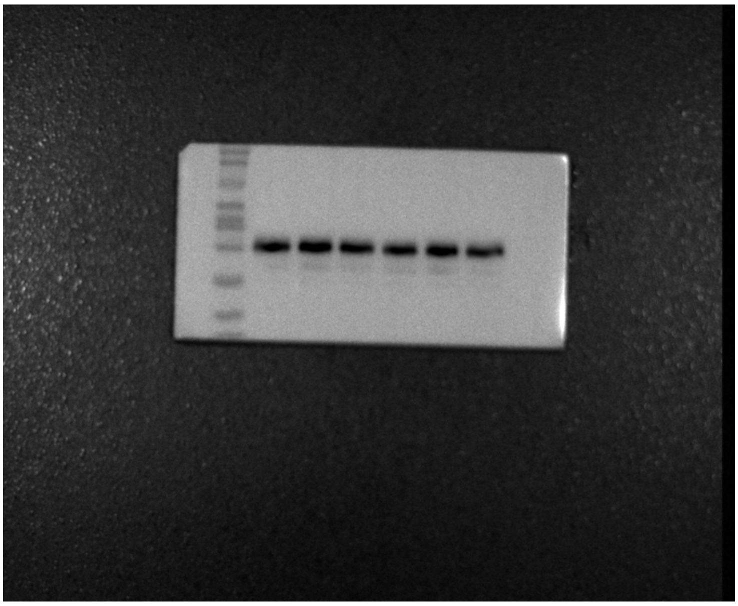

Figure 7. Effect of chlorogenic acid (CGA) on nuclear factor-κB (NF-κB) activation. The NF-κB pathway p-P65 protein expression was analyzed in BMECs via Western blot.

Note: Marker, NC, LTA, LTA+CGA (25 ug/mL), LTA+CGA (50 ug/mL), LTA+CGA (100 ug/mL), Dexamethasone (positive control).

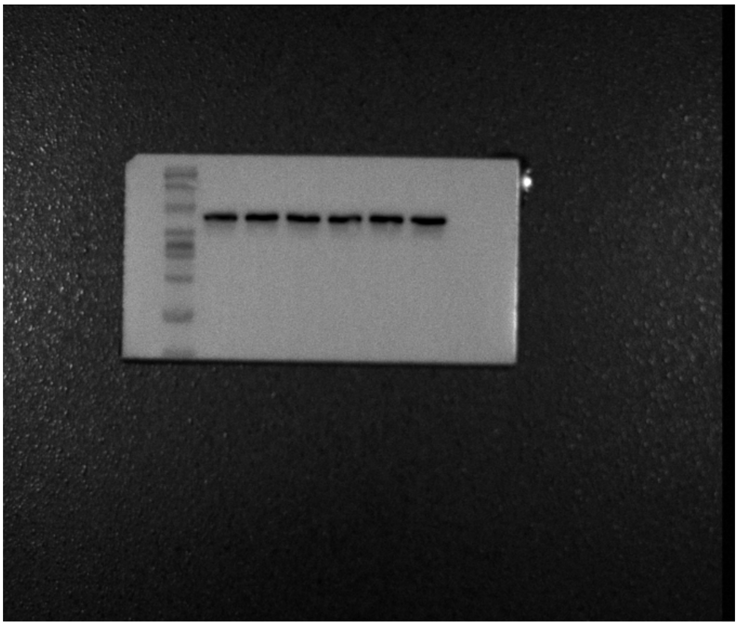

Figure 7. Effect of chlorogenic acid (CGA) on nuclear factor-κB (NF-κB) activation. The NF-κB pathway P65 protein expression was analyzed in BMECs via Western blot.

Note: Marker, NC, LTA, LTA+CGA (25 ug/mL), LTA+CGA (50 ug/mL), LTA+CGA (100 ug/mL), Dexamethasone (positive control).

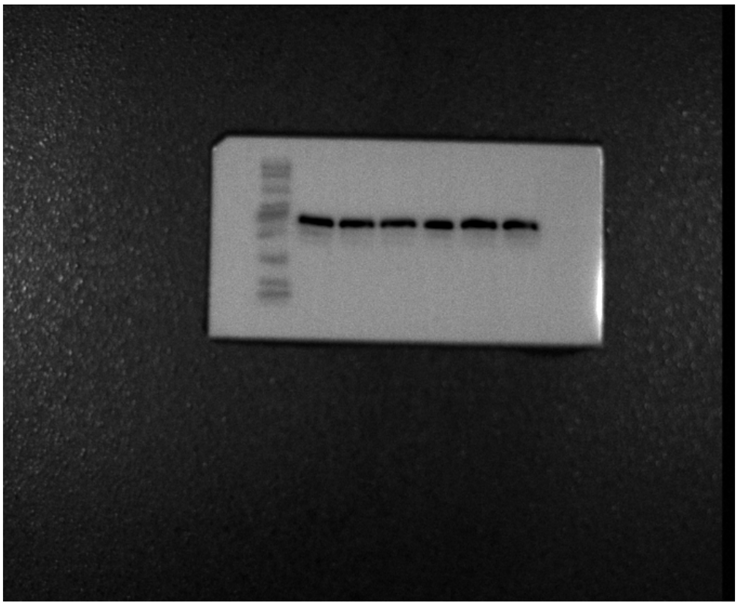

Figure 7. Effect of chlorogenic acid (CGA) on nuclear factor-κB (NF-κB) activation. β-actin was used as a reference control.

Note: Marker, NC, LTA, LTA+CGA (25 ug/mL), LTA+CGA (50 ug/mL), LTA+CGA (100 ug/mL), Dexamethasone (positive control).
